# Knock-down of Hdj2/DNAJA1 co-chaperone results in an unexpected burst of tumorigenicity of C6 glioblastoma cells

**DOI:** 10.18632/oncotarget.7872

**Published:** 2016-03-03

**Authors:** Darya A. Meshalkina, Maxim A. Shevtsov, Anatoliy V. Dobrodumov, Elena Y. Komarova, Irina V. Voronkina, Vladimir F. Lazarev, Boris A. Margulis, Irina V. Guzhova

**Affiliations:** ^1^ Institute of Cytology of Russian Academy of Sciences, St. Petersburg 194064, Russia; ^2^ First I.P. Pavlov State Medical University of St. Petersburg, St. Petersburg 197022, Russia; ^3^ Institute of Macromolecular Compounds of the Russian Academy of Sciences, St. Petersburg 199004, Russia

**Keywords:** glioblastoma, Hdj2, Hsp70, tumorigenicity, metastases

## Abstract

The chaperone system based on Hsp70 and proteins of the DnaJ family is known to protect tumor cells from a variety of cytotoxic factors, including anti-tumor therapy. To analyze whether this also functions in a highly malignant brain tumor, we knocked down the expression of Hsp70 (HSPA1A) and its two most abundant co-chaperones, Hdj1 (DNAJB1) and Hdj2 (DNAJA1) in a C6 rat glioblastoma cell line. As expected, tumor depletion of Hsp70 caused a substantial reduction in its growth rate and increased the survival of tumor-bearing animals, whereas the reduction of Hdj1 expression had no effect. Unexpectedly, a reduction in the expression of Hdj2 led to the enhanced aggressiveness of the C6 tumor, demonstrated by its rapid growth, metastasis formation and a 1.5-fold reduction in the lifespan of tumor-bearing animals. The *in vitro* reduction of Hdj2 expression reduced spheroid density and simultaneously enhanced the migration and invasion of C6 cells. At the molecular level, a knock-down of Hdj2 led to the relocation of N-cadherin and the enhanced activity of metalloproteinases 1, 2, 8 and 9, which are markers of highly malignant cancer cells. The changes in the actin cytoskeleton in Hdj2-depleted cells indicate that the protein is also important for prevention of the amoeboid-like transition of tumor cells. The results of this study uncover a completely new role for the Hdj2 co-chaperone in tumorigenicity and suggest that the protein is a potential drug target.

## INTRODUCTION

Glioblastoma multiforme is the most aggressive of brain tumors, with a very poor prognosis. The median survival of patients with a standard of care therapy that currently includes surgical removal of the tumor with subsequent radio- and chemotherapy with temozolomide, does not exceed 14.6 months [1, 2]. Therefore, the development of novel therapeutic approaches to glioblastoma treatment and the discovery of potential drug targets are urgently required.

Molecular chaperones, especially the 70-kDa heat-shock protein (Hsp70), play an important role in the processes of protein folding, signal transduction and general response to stress factors [3, 4]. The expression of Hsp70 in cancer cells is associated with an increase in their proliferation and poor prognosis in patients with various cancer localizations [5, 6]. A high level of Hsp70 was found in patients with acute myeloid leukemia, breast cancer, endometrial carcinoma, colorectal adenocarcinoma and was correlated with a decreased overall survival [7–10]. Central nervous system neoplasms, including low- and high-grade gliomas, are also associated with the over-expression of Hsp70 and Hsp90 chaperones [11]. The involvement of Hsp70 in tumor development occurs at all steps of the tumor progression, but its role in cancer invasion and metastasis has received much attention in the past decade [12, 13]. The chaperone has been implicated in cancer cell motility [14, 15], which might be linked to the ability of the membrane-bound Hsp70 to influence the spread of the distant metastasis [16].

The chaperone machinery has been extensively studied within the last 15–20 years. It was clearly demonstrated that Hsp70 activity is assisted by J-proteins that exhibit important intracellular functions, such as binding to the unfolded client protein and its delivery to Hsp70, which stimulates Hsp70 ATPase activity [17]. The J-proteins belong to the largest and the most diverged group of molecular chaperones that contains a conserved J-domain, which was defined first in the canonical *Escherichia coli* chaperone, DnaJ [18]. The family consists of 49 members and is divided into three groups, depending on the localization of the J-domain within a protein molecule. Type I DNAJ proteins (DNAJA, four members in humans) consist of a N-terminal J-domain, a glycine-/phenylalanine- (G/F) rich region, a cysteine-repeat (Cys-repeat) region and a largely uncharacterized C-terminus, whereas type II DNAJ proteins (DNAJB, 13 members) lack the Cys-repeat region and have an extended G/F rich region. Type III DNAJs (DNAJC, 32 members) differ substantially from type I and type II DNAJs as they lack the G/F and Cys-repeat regions and the J-domain can be situated anywhere within the protein [19–21].

Although the role of Hsp70 in cancer development is well documented, data concerning the function of its most abundant cellular co-chaperones, Hdj1 (DNAJB1) and Hdj2 (DNAJA1), in the process remain elusive. In this study, we chose the intracranial C6 rat glioblastoma model and found that the depletion of Hsp70 (HSPA1A) via lentiviral constructs delayed tumor growth, whereas the inhibition of Hdj1 resulted in no changes in tumor development. Surprisingly, knock-down of Hdj2 caused an increase in C6 tumor growth and strongly reduced animal survival. The *in vitro* data led us to conclude that a reduction in Hdj2 might lead to the pronounced enhancement of C6 cells tumorigenicity, particularly their mobility and invasiveness.

## RESULTS

### shRNA-mediated knock-down of chaperone gene expression

**Figure 1:**
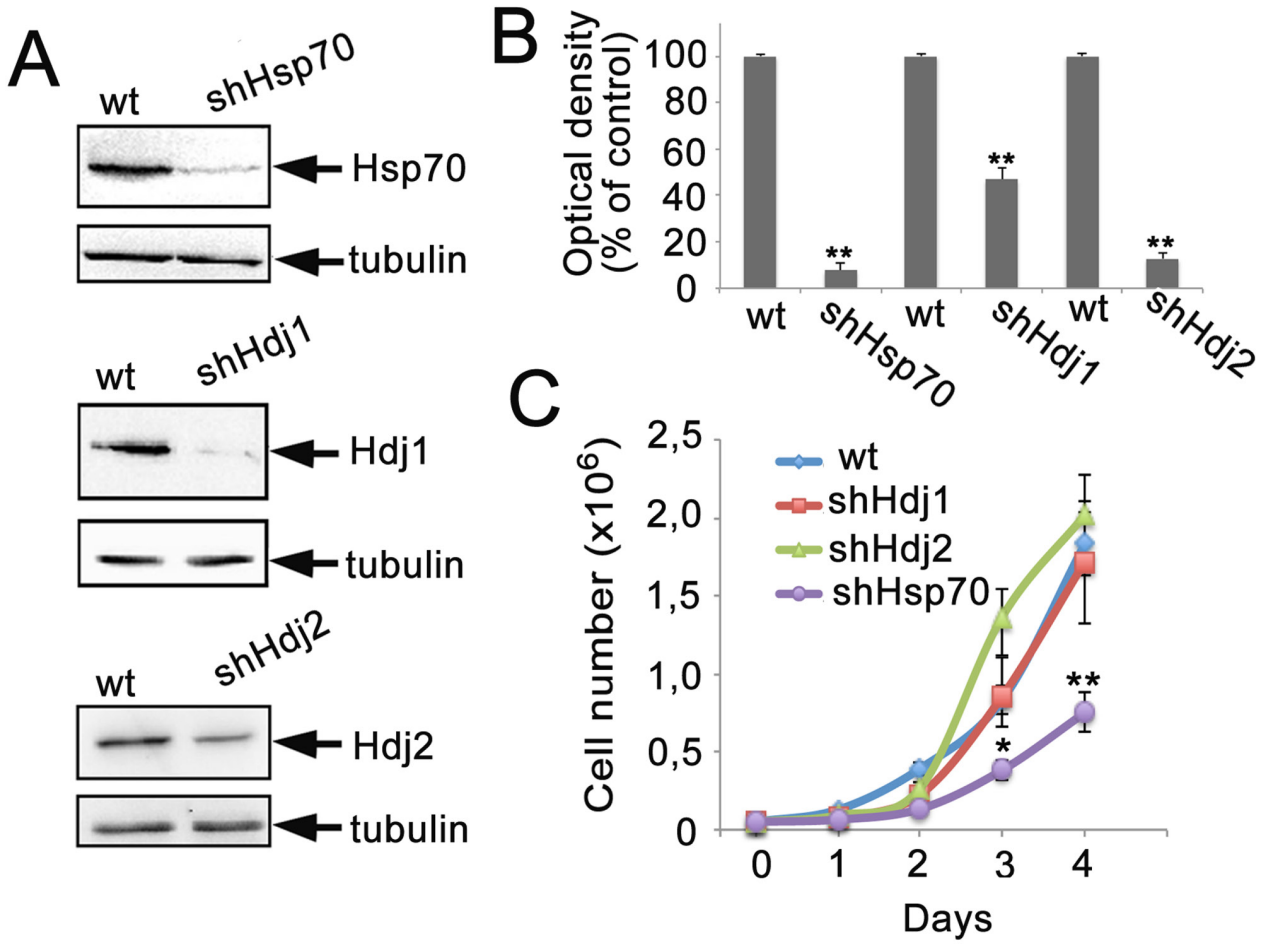
The reduction in expression of Hsp70, Hdj1 and Hdj2 chaperones in C6 rat glioma cells The C6 cells were infected with lentivirus-encoded shRNA directed against sequences in HSPA1A (Hsp70), DNAJB1 (Hdj1) and DNAJA1 (Hdj2) chaperones. **A.** Representative Western blot for C6 cell lines: C6-wt, C6-shHsp70, C6-shHdj1 and C6-shHdj2. The lysates of cells of the lines indicated were subjected to polyacrylamide gel electrophoresis and the membranes obtained after blotting were stained with the appropriate antibodies. **B.** The intensity of bands in A was estimated with the use of Image J Software. Data of two independent experiments were calculated. **C.** Growth rates of C6-wt, C6-shHsp70, C6-shHdj1 and C6-shHdj2 cell sub-lines. Statistical significance is indicated as *P < 0.05 and **P < 0.001.

To explore the influence of a particular chaperone level on tumor development, we created three C6-based cell lines, which constitutively expressed shRNA to Hdj1, Hdj2 or Hsp70. These cell lines were designated as C6-shHdj1, C6-shHdj2 and C6-shHsp70, respectively. The inhibition of gene expression in these cell lines was established by Western blotting and validated by Image J software. Compared to the control the concentrations of the chaperones were reduced as follows: shHdj1 by 92.3%, shHdj2 by 53.2% and shHsp70 by 87.2% (Figure 1A, 1B).

All obtained cell lines showed slight but stable changes in cell morphology (data not shown). The C6-shHdj1 cells were very similar to those of C6-wt, but had fewer side protrusions; C6-shHdj2 cells appeared to become more roundish and less attached to the substrate, with a considerable fraction of floating living cells, needle-like protrusions and a large number of leading edges in the culture. The C6-shHsp70 cells appeared rather elongated and fibroblast-like.

We measured the growth rate and plotted all four growth curves for 4 days, starting from 5 × 10^4^ cells per mL and found that three cell C6 sub-lines: C6-wt, C6-shHdj1 and C6-shHdj2 showed practically indistinguishable growth rates, but C6-shHsp70 grew slower and reached confluence later (Figure 1C).

### The knockdown of chaperones affects glioblastoma growth *in vivo*

To estimate the tumor growth following chaperone knockdown, we used magnetic resonance imaging. For these experiments C6-wt, C6-shHdj1, C6-shHdj2 and C6-shHsp70 cells were inoculated into the brain of rats, constituting four groups of 15 animals in each group. On the twentieth day following the injection, three animals from each group were anesthetized and were assessed using the high-field MR scanner. Two animals from each group were sacrificed, and the brains were extracted and fixed in 4% formalin for histological analysis. Another 10 rats remaining in each group were used to evaluate the survival rate after tumor inoculation.

**Figure 2:**
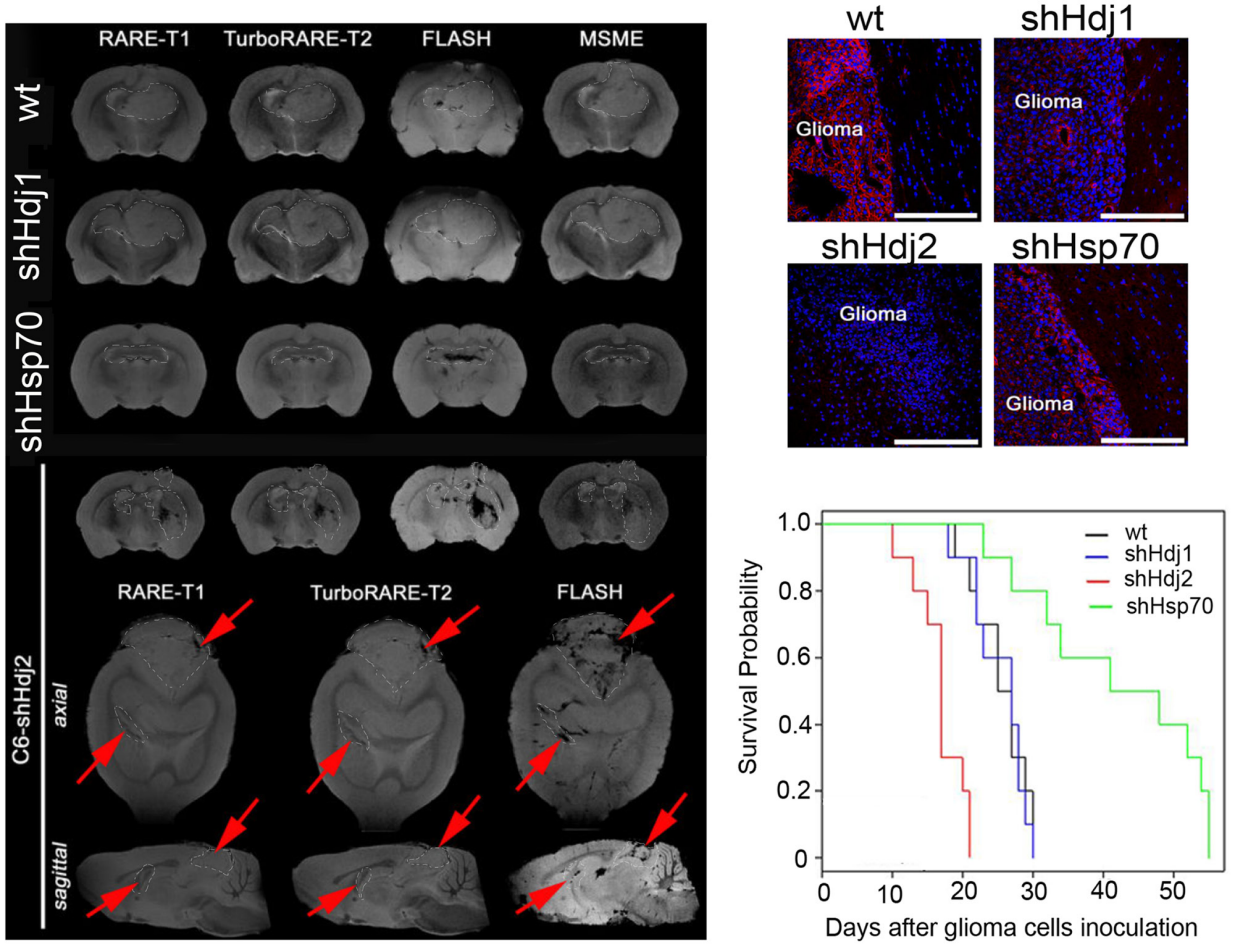
Knockdown of Hsp70 in C6 cells delays tumor growth and down-regulation of Hdj2 increases tumor aggressiveness and distant tumor lesion formation *in vivo* **A.** Magnetic resonance brain imaging of C6-wt, C6-shHsp70, C6-shHdj1 and C6-shHdj2 glioblastoma-bearing animals on the 20-th day following tumor cell inoculation. On the additional axial and saggital MR scans for the C6-shHdj2 tumor, the leptomeningeal metastases are marked by red arrows. The MR images were obtained using FLASH, RARE-T1, TurboRARE-T2 and MSME regimes. **B.** Histological images of the tumor sections of C6-wt, C6-shHsp70, C6-shHdj1 and C6-shHdj2 glioma-bearing animals. F-actin was stained with rhodamine-phalloidin (red), and nuclei with DAPI (blue). Scale bar, 300 μM. **C.** Cumulative proportional survival of animals inoculated with C6-wt, C6-shHsp70, C6-shHdj1 and C6-shHdj2 cells, Kaplan-Meier graphs (N = 10 per group).

The C6-wt tumor was represented as a large tumor with a hypotensive core on the T2-weighted images, with significantly increased R2 and R2* coefficients of relaxivity (Figure 2A). The pattern of C6-shHdj1 did not differ from that in animals with wild-type tumors. The down-regulation of Hsp70 in C6-shHsp70 cells resulted in a significant delay in tumor growth in comparison to C6-wt or C6-shHdj1 tumors. The border of the C6-shHsp70 glioblastoma was clearly delineated from the surrounding normal brain tissues. Intriguingly, when the C6-shHdj2 glioblastoma-bearing animals were assessed, we observed numerous leptomeningeal metastases in all tested animals (Figure 2A). We found brain-stem C6-shHdj2 metastases that were highly invasive into the surrounding normal tissue. The data from MR images were supported by the histological analysis of the tumor sections (Figure 2B). In a group of inoculated C6-shHdj2 animal cells, the tumor was highly invasive into the brain tissue. The dynamics of glioblastoma growth influenced animal survival (Figure 2C). The overall survival period for C6-wt animals was 25.5 ± 3.8 days. Down-regulation of Hdj1 did not influence the survival rate, which was 25.4 ± 3.9 days, whereas Hsp70 inhibition increased the survival rate by almost two-fold (up to 42.1 ± 12.3 days). On the contrary, knock-down of Hdj2 resulted in a decrease in the survival rate to 16.8 ± 3.5 days, whereas the culture growth rate remained the same as that of wild type C6. The differences between survival rate in experimental groups with animals with C6-wt tumors and with C6-shHsp70 or with C6-shHdj2 tumors were statistically significant (p<0.01).

### C6 cells with Hdj2 knock-down detached more readily from the initial population and formed new growth foci *in vitro*

**Figure 3:**
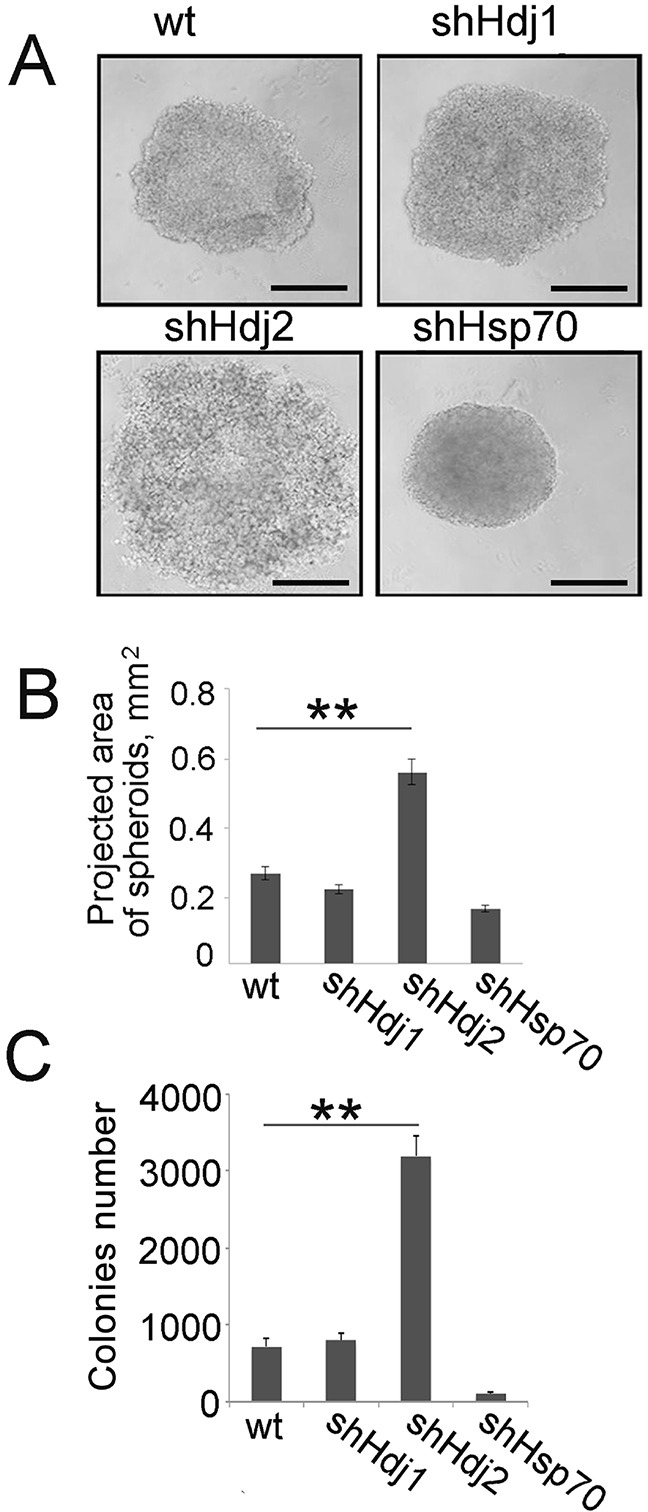
Cells of C6 glioblastoma with down-regulated Hdj2 expression demonstrate an increased ability to detach from the initial cell population and to inhabit a new area **A, B.** Cells of C6-wt, C6-shHsp70, C6-shHdj1 and C6-shHdj2 were incubated in wells of a U-shaped 24-well microplate for 24 h and photographed. Scale bar 100 μm (A). The area of the spheroid projections was calculated using Image J software. The values of five measurements were averaged and the SE was determined. Statistical significance is indicated as *P < 0.05; **P < 0.001. **C.** Cells of C6-wt, C6-shHsp70, C6-shHdj1 and C6-shHdj2 were seeded into wells of 24-well plates and allowed to attach to the bottom for 4 h and after replacing the medium, cells were incubated for the next 18 h. The culture medium with floating cells was collected and transferred to wells of six-well plates and left for further 24 h. Colonies formed by attached cells were calculated using phase-contrast microscopy. The data for three independent experiments are presented.

Since tumors formed by C6-shHdj2 cells did not have a border with the surrounding tissue, we suggested that the ability of these cells to form intercellular contacts is reduced. To analyze this, we formed spheroids using 10^5^ cells of each C6 sub-line. Spheroids created by C6-shHdj2 cells were three-fold larger and had a spongy, crumbly structure, suggesting that the cells were not connected tightly to each other and could easily dissociate from the spheroids (Figure 3A, 3B). Notably, shHsp70 cells formed more compact and dense spheroids than control cells.

Next, we checked whether C6 cells that are depleted of chaperones have a distinct ability to separate from the initial tumor and to attach to a new substrate. To test whether the floating cells in the C6-shHdj2 population are viable and can reattach to the substrate, we collected culture supernatants from C6-wt, -shHdj1, -shHdj2 and -shHsp70 cells after 18 h of cultivation and reseeded them to fresh wells of a 24-well plate. After 24 h, we counted the number of colonies that arose from floating cells. (Figure 3C). The number of colonies was much higher in C6-shHdj2 cells and reached 3274 ± 184, whereas the number for C6-wt cell colonies was 695 ± 116. The C6-shHsp70 cells demonstrated the lowest ability to disseminate and the number of colonies was 88 ± 64 (Figure 3C).

**Figure 4:**
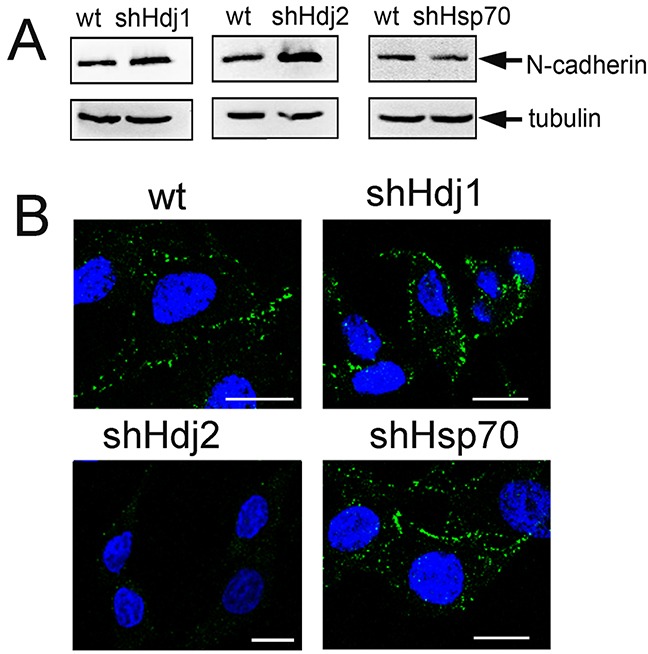
Knock-down of Hdj2 in C6 glioblastoma cells leads to the enhancement of N-cadherin intracellular amount and to its redistribution **A.** Analysis of N-cadherin in C6-wt, C6-shHsp70, C6-shHdj1 and C6-shHdj2 cells via Western blotting. An anti-tubulin antibody was used as a loading control. **B.** Confocal microscopy of C6-wt, C6-shHsp70, C6-shHdj1 and C6-shHdj2 cells with the surface staining of N-cadherin (green). Nuclei were stained with DAPI (blue). Scale bar = 5 μm.

The spongy structure of C6-shHdj2 spheroids and the enhanced ability of the cells to detach from the primary population and to form new distant cellular growth sites might be due to a loss of specific adhesion molecules such as cadherins, which are responsible for cell–cell adhesion [22]. The C6 cells do not express E-cadherin, that was confirmed by Western blotting (Supplementary Figure S1, Supplementary Materials), but they possess a high level of N-cadherin expression (Figure 4A). Importantly, the content of N-cadherin in C6-shHdj2 cells was higher by 71% than in C6-wt cells, which contradicts their propensity to detach from a substrate shown previously. We stained C6 cells of four sub-lines with an antibody against N-cadherin and found that despite its elevated content in C6-Hdj2 cells, N-cadherin does not localize to the plasma membrane as was shown for C6-wt, -shHdj1, and -shHsp70 cells (Figure 4B).

### The down-regulation of Hdj2 leads to an increased invasiveness of C6 cells

**Figure 5:**
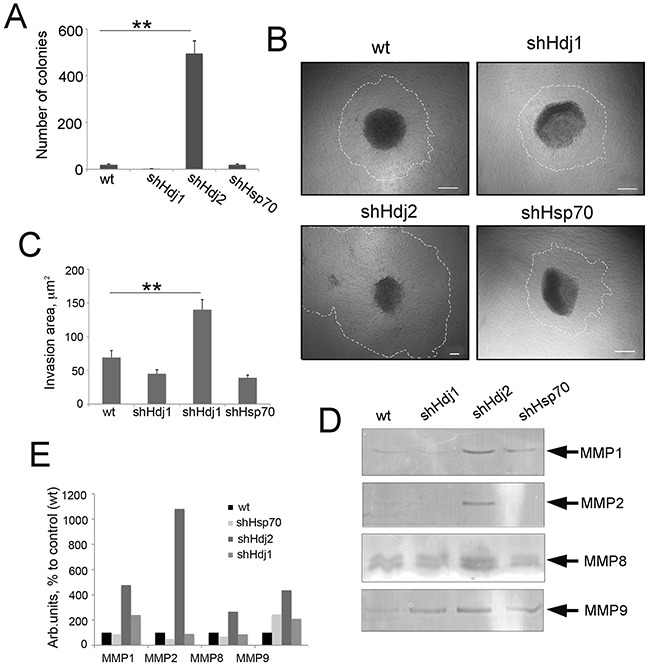
The knock-down of Hdj2 expression in C6 glioblastoma cells causes an increased ability for invasion as demonstrated by cell growth in Matrigel and metalloproteinase expression **A.** Cells of C6-wt, C6-shHsp70, C6-shHdj1 and C6-shHdj2 were seeded into Boyden chambers with a membrane with pores of 8 μm. Twenty h later, cells on the plate bottoms were fixed and stained and the number of the colonies was calculated. Data of three independent experiments are presented. **B.** Spheroids of C6-wt, C6-shHsp70, C6-shHdj1 and C6-shHdj2 lines were placed into Matrigel, incubated overnight and photographed. Scale bar 100 μm. **C.** The invasion area was estimated as a residual between the total spheroid area with cells growing around (traced area) and the area of spheroid body itself determined using Image J Software. C. Activity of MMPs-1, -2, -8 and -9 in the culture medium of C6wt, C6-shHsp70, C6-shHdj1 and C6-shHdj2 cells as measured with the use of zymography. **D.** Quantitative analysis of MMP activity was performed using Image J software.

The friable structure of spheroids formed by cancer cells are prerequisites for their increased invasiveness. To investigate the invasion properties of the C6 chaperone knock-down sub-lines, we performed a standard transwell migration assay. We seeded the cells of interest in the upper part of the Boyden chamber in serum-free media and observed their migration to the lower well, containing 10% FBS. Firstly, we determined the percentage of cells that migrated to the lower side of the Boyden chamber membrane, and this was practically the same in all four cell lines tested (65%). We then counted the colonies that formed on the bottom of the wells (Figure 5A): C6-shHdj2 formed significantly more colonies (495 ± 57) on the bottom of the well than the other cell lines. Cells of C6-shHsp70 line formed more colonies (29 ± 11) than C6-wt (5 ± 4) and C6-shHdj1 (1 ± 1), which can be explained by elevated adhesion properties of the cell line, which allow it to migrate through the membrane more successfully. Therefore, the decreased adhesion of C6-shHdj2 cells was nevertheless sufficient for their migration into the transwell.

To compare the invasive ability of C6 cells with down-regulated Hsp70, Hdj1 and Hdj2 chaperones, we also formed spheriods as described above, embodied them into Matrigel and incubated for 24 h. During this time, cells liberated from the spheroid body grew around the spheroids (Figure 5B) and we estimated the invasion area as a residual between the total spheroid area with cells growing around and the area of spheroid body itself (Figure 5C). The invasion area of the spheroids formed by C6-shHdj1 cells were similar to that of C6-wt spheroids and comprised 44.9 ± 6.7 and 69.6 ± 6.9 μm^2^, respectively, whereas the invasion zone of C6-shHsp70 spheroids was smaller and was 37.9 ± 6.8 μm^2^. The spheroids formed by C6-shHdj2 cells occupied the highest invasion area, which extended to 139.6 ± 11.6 μm^2^. Additionally, we observed new small structures near larger ones, which probably detached from “mother” spheroids during incubation. (Figure 5B, 5C).

Another factor that promotes the invasion of cancer cells is the activity of matrix metalloproteinases (MMPs) [23], and we measured their activity in cells with a modified expression of the chaperones. The data from zymography of the culture medium collected from all C6 cell cultures, show that the Hdj2 knock-down cells indeed, had a much more active MMP-1, MMP-2, MMP-8 and MMP-9 than C6-wt cells. The activity of these MMPs was 4.8-fold, 2.7-fold and 4.4-fold higher, respectively, in C6-shHdj2 than in C6-wt cells (Figure 5D, 5E). The knock-down of Hsp70 also caused an increase in MMP-9 activity (3.4-fold compared to C6-wt cells), although other MMPs were shown to be less active. The down-regulation of Hdj1 resulted in the hyperactivation of MMP-1 (2.4-fold) and MMP-9 (2.1-fold), but the activity of MMP-2 and MMP-8 did not differ from that of C6-wt cells (Figure 5D, 5E).

### The knock-down of Hdj2 in C6 cells increases their migration ability

**Figure 6:**
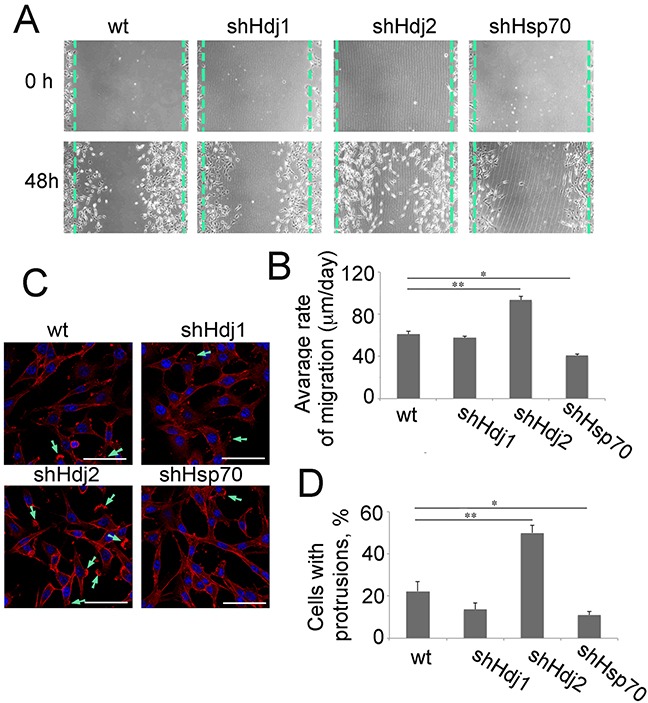
Downregulation of Hdj2 in C6 cells modulates the ability for cell migration **A.** Glioblastoma cells of C6-wt, C6-shHsp70, C6-shHdj1 and C6-shHdj2 lines were cultured in six-well plates. A straight scratch was made in individual wells with a 200-μL pipette tip, and the wound was photographed under a microscope after 48 h of incubation. **B.** The distance that each individual cell migrated within the wound area during 48 h of cultivation was measured using Image J software. **C.** Cells of C6-wt, C6-shHsp70, C6-shHdj1 and C6-shHdj2 were stained with Phalloidin-Rhodamine and images were taken with a confocal microscope. Protrusions stained with Phalloidin-Rhodamine are indicated with green arrows. **D.** The number of cells with a protrusion was calculated for each C6 sub-line.

The analysis of the cell migration activity of C6-wt, -shHdj1, -shHdj2 and -shHsp70 was performed using the scratch and wound-healing assays (Supplementary Figure S2 in Supplementary Materials). Using both methods, we showed that the knockdown of Hdj1 does not influence the migration ability of C6 cells, whereas the motility of C6-shHsp70 cells was significantly reduced, reminiscent of their stunted growth in cell culture (Figure 1B) and in the rat brain (Figure 2A). Importantly, C6-shHdj2 cells that demonstrated a similar growth rate to C6-wt cells could form colonies in the center of a scratch without contacts with the growing edge or with each other. Simultaneously, detached floating cells were found in the culture supernatant, demonstrating that C6-Hdj2 cells were disseminated far beyond the culture edge (Figure 6A). The mean speed of migrated C6-shHdj2 cells to the scratch area was 93.5 ± 3.3 μm/day, whereas this value for C6-wt and C6-shHdj1 cells was 61.2 ± 2.9% and 57.7 ± 1.7 μm/day, respectively. The C6-shHsp70 cells colonized the scratched area slower, at a rate of 40.9 ± 1.2 μm/day (Figure 6B). Similar results were obtained via the wound healing assay (Supplementary Figure S2 in Supplementary Materials).

Cell migration is a process that requires the formation of actin-based membrane protrusions on the leading edge [24]. To analyze the state of the actin cytoskeleton in modified C6 cells, we stained them with rhodamine-phalloidin. Cells of all C6 sub-lines except for C6-shHsp70 contained actin-positive protrusions, but the number of cells having these specific foci was maximal in the population of C6-shHdj2 cells, where 50.0 ± 3.5 % of cells had protrusions, whereas in C6-wt, C6-shHdj1 and C6-shHsp70 cells, this amount was 22.3 ± 4.5%, 13.6 ± 3.2% and 11.1 ± 1.5%, respectively (Figure 6C, 6D).

The effect of Hdj2 suppression extends not only C6 cells. We performed similar experiments with shRNA interference targeting B16 mouse melanoma cells and found that their migration rate and the expression of two metalloproteinases 2 and 9 are strongly elevated after depletion of the co-chaperone (Supplementary Figure S3 in Supplementary Materials).

## DISCUSSION

The major stress protein Hsp70 is over-expressed in a plethora of human tumors, probably because cancer cells demonstrate rapid and strong metabolism and enhanced protein synthesis that require a higher level of cellular chaperones to regulate the correct folding of nascent polypeptides and their transport to target cellular organelles [25]. Another reason for the increase in Hsp70 in tumor cells is the need for homeostatic mechanisms based on the chaperone, which confer efficient tolerance to the effects of chemical and biological stressors such as reactive oxygen species or tumor necrosis factor [26].

The function of Hsp70 is aided by DnaJ-like proteins, which are called co-chaperones and which assist the master chaperone to recognize the protein substrate and to fold it correctly; it is clear that the expression of these proteins should be enhanced in tumors that over-express Hsp70. The analysis of Hdj1 expression in ovarian, endometrial and uterine tumors from the City Oncology Clinic (St.Petersburg, Russia) led us to conclude that the co-chaperone content of tumor cells was high in benign tumors and was even higher in malignant ones [27]. On the contrary, expression of another member of the family of DnaJ class proteins, Hdj2, was five-fold lower in pancreatic cancer cells taken from patients [28]. These examples highlight the lacunae in our current knowledge of the role of DnaJ-like proteins in human cancer and require further investigation.

To elucidate the role of two co-chaperones, Hdj1 and Hdj2, in the pathology of C6 rat glioblastoma, we generated three sublines of these cells that contained the knock-down of expression of the master chaperone Hsp70 and both co-chaperones. The C6-wt, -shHdj1 and -shHdj2 cells grew at a similar rate, whereas the growth of C6-shHsp70 cells was inhibited (Figure 1). Previously, down-regulation of Hsp70 was shown to cause tumor growth arrest in gastric cancer cells [29] and to promote G2/M arrest in HeLa cells [30]. The depletion of Hsp70 was also shown to sensitize myc-overexpressing lymphoid cancer cells to drug-induced apoptosis [31], moreover, inhibition of Hsp70 synthesis by transfection of antisense Hsp70 cDNA results in massive death of human breast cancer cells [32] and caused senescence in cells transformed with RAS, Her2 or PIK3CA oncogenes [33].

Next, we explored the growth characteristics of modified tumor cells *in vivo*. Cells of all four sub-lines were grafted into rat brains and we found that the tumors formed by C6-wt and C6-shHdj1 grew at the same rate, whereas C6-shHsp70 tumors demonstrated delayed progression in concordance with growth arrest of in a murine glioblastoma xenografts [34]. Notably, we found distant tumor lesions on MR images of rat brains with C6-shHdj2 tumors (Figure 2A). The results of the histological analysis, which showed that these tumors had no clear border from the surrounding brain tissue support the suggestion that knock-down of Hdj2 might cause the process of metastasis.

Metastasis formation is a complex process that starts from the detachment of solitary tumor cells from the body of a primary tumor and the invasion of lymphatic vessels and capillaries [35]. To analyze whether C6-shHdj2 cells demonstrate typical features of a metastasizing tumor, we performed *in vitro* experiments that aimed to measure the ability to detach and to form colonies. Firstly, we found that C6-shHdj2 cells can dissociate from the original tumor cell monolayer more readily than the cells of the other three sub-lines (Figure 3). We believe that this behavior of C6-shHdj2 cells correlates with the first stage of a tumor metastasis cascade [36].

Another feature of metastasizing cancer cells is the loss of intercellular contacts that are provided by cadherins shown to be tissue-specific [37]: E-cadherin is specific for endothelial tissues, N-cadherin was found in the brain and P-cadherin is present in the placenta. In most epithelial cancers, the loss of E-cadherin leads to the transition to a mesenchymal, migratory, and invasive cell phenotype, a process called the epithelial-to-mesenchymal transition [38]. Although C6 glioblastoma cells do not express E-cadherin (Supplementary Figure S1), the level of N-cadherin was found to be much higher in C6 cells with down-regulated Hdj2 than in cells of other sub-lines (Figure 4A). The increase in N-cadherin expression was observed at an early stage in more invasive and less differentiated breast cancer cell lines that lacked E-cadherin expression [39] and in squamous carcinomas [40]. It was proposed that N-cadherin contributes directly to the invasive phenotype. Using confocal microscopy, we found that N-cadherin disappeared from the cell surface of C6-shHdj2 cells, which might explain the weak contacts between these cells (Figure 4B). To our knowledge, this is the first observation of the redistribution of N-cadherin in highly tumorigenic cells.

The surface expression and function of cell adhesion molecules like N-cadherin are modulated by endocytosis and recycling [41, 42] and causes by the defect observed with loss of function of Rab GTPases [43]. In Rab5 deficient neurons an increased surface expression of N-cadherin and an altered N-cadherin distribution between the cell body and the processes was observed. Rab7 deficient cells had increased total cellular level of N-cadherin, which is consistent with a decrease lysosomal degradation of protein. Rab1 deficiency caused an accumulation of N-cadherin in cytoplasmic vesicles [42, 44]. As a co-chaperone and apart its chaperonic function Hdj2 is able to bind cellular proteins and Rabs proteins could be among its partners. To favor of this suggestion is the fact that Hdj2 diminishes extracellular Hsp70 transport from cancer cells (Shevtsov et al., paper in preparation). The future proteomic analysis of Hdj2-binding proteins could unveil this question.

Invasion is a critical step in the metastatic cascade and to measure this property of C6 cell sub-lines, we used a morphological approach, using cell-spheroid growth in a matrigel. This novel method recently described by Chandrasekaran and coauthors (2015) was applied to the analysis of HCT116 colon cancer cells that completely lacked the expression of the PTEN tumor suppressor gene [45]. The results of this assay showed that spheroids formed by C6-shHdj2 cells with the extensions formed by the cells liberated from spheroid body, occupy a 2.5-fold greater area than those of the other cells (Figure 5A, 5B). In a view of data of Chandrasekaran et al it worth mentioning that the knock-down of two completely unrelated genes and their products stimulate the burst of cancer metastasis. Metalloproteinases also serve as important markers for the final stage of the metastatic process [46]. The results of zymography showed that C6-shHdj2 cells possess highly active MMP-1 and -2 (Figure 5D, 5E), whose activation was already demonstrated in a variety of human cancers [47].

Metastatic cells possess the enhanced motility associated with the proper function of the actin cytoskeleton and we studied the latter in C6 cell sub-lines. Using the scratch test, we found that the rate of migration of C6-shHdj2 cells was 1.5-fold higher than that of other cells (Figure 6A, 6B). Similar data were reported for the behavior of multiple myeloma cells that possessed a knock-down in expression of the SPRY2 gene [48] and after pharmacological inhibition of expression of the urokinase-type plasminogen activator in human breast carcinoma cells [49]. The results of the scratch-test were confirmed by the wound-healing assay, which showed that C6-shHdj2 cells migrated to the wound area faster than cells of other sub-lines (Supplementary Figure S2); again, this agrees well with previous data of Zhang et al. [49] for cells that acquire a more metastatic phenotype. Indeed, actin distribution analysis confirmed that C6-shHdj2 cells attain additional protrusions that can be stained with phalloidin compared to C6 cells of other sub-lines (Figure 6C, 6D). This signifies that the cells with a lower content of the Hdj2 co-chaperone have better developed actin bundles, thus enabling them to reach the site of metastasis formation more efficiently [see 50 for review].

Taken together, these data show that the depletion of Hdj2 in C6 glioblastoma cells leads to a highly aggressive tumor phenotype with all attributes of actively metastasizing cells, including detachment from the primary tumor, increased motility and invasion characteristics.

The role of J-domain-containing proteins in cancer development is obscure; Hdj2 expression was five-fold lower in samples from patients with pancreatic cancer [51, 28]. The elevated expression of Hdj2 in glioblastomas led to radiosensitization, whereas its low expression was linked to radioresistance [52]. The members of the B-subfamily of J-proteins, HLJ1 (*DNAJB4*) and MRJ (DNAJB6), demonstrated a similar behavior to that of Hdj2 shown in this study.

The positive and negative regulation of J-domain- proteins was recently reported by two groups. In one study, MRJ was found to be in a triple complex with Hsp70 and a urokinase-type plasminogen activator receptor; the knock-down of MRJ (or Hsp70) caused the dissociation of the complex and inhibited the expression of MMP-2 and MMP9 [53]. These data contrast with the findings of Mirta and coauthors [54], who demonstrated that the upregulation of MRJ synthesis inhibited migration, invasion and tumorigenesis in breast cancer.

The other member of the DNAJB family is HLJ1, which is a potential marker of colorectal cancer-cell metastasis. The expression of HLJ1 was lower in samples from patients with highly metastatic colorectal cancer than in lowly metastatic samples. Moreover, patients with a high level of HLJ1 had a better overall survival rate than patients with a low rate [55]. The high expression of HLJ1 in non-small-cell lung carcinomas inhibited proliferation, growth, motility and invasion and promoted apoptosis [56].

It appears that Hsp70 master chaperone and its assistants belonging to the DNAJ family may play opposite roles in a cancer cell: the co-chaperones tune the cancer cell mechanism to low progression, whereas Hsp70 protects the cell against anti-tumor therapy.

Hypothetically, Hdj2 can influence the tumor-cell phenotype, due to its membrane-associated localization, which can be lost upon heat shock [57] or farnesyl-transferase inhibition [58]. Evidence for its function derived from the results of high throughput screening, in which Hdj2 was found to associate with integrin-linked kinase (ILK) [59]. Since ILK mediates integrin signal transduction, Hdj2 can affect the cell architecture, adhesion and migration along integrin substrates.

In conclusion, Hdj2 functions as a co-chaperone of Hsp70; however, recent data and our results suggest that the former can also play individual role in tumor progression, which as shown here, is very important.

## MATERIALS AND METHODS

### Cells

The C6 glioblastoma and HEK-293T cells were provided by the Russian Cell Culture Collection from the Institute of Cytology of the Russian Academy of Sciences (RAS) (St. Petersburg, Russia). The C6 cells were grown in DMEM/F12 medium supplemented with 10% fetal bovine serum (FBS), 2 mM L-glutamine and antibiotics (100 U/mL penicillin G and 0.1 mg/mL streptomycin). Cells were grown in a CO_2_-incubator with 6% CO_2_ and 90% humidity. Viability was determined by 0.4% trypan blue exclusion. All culture reagents were purchased in PanEco (Russia).

Vector plasmids for knock-down of Hsp70 and its co-chaperones were purchased from GE Dnarmacon, CO, USA: clones TRCN0000008513 – shRNA to HSPA1A (Hsp70), mature antisense: TTGATGCTCTTGTTCAGGTCG; TRCN0000008791 – shRNA to DNAJB1 (Hdj1), mature antisense: TTCATCTTCTTGGTACAGCCG; TRCN0000156034 – shRNA to DNAJA1 (Hdj2), mature antisense: ATGCTTGACAATCTGACCTGG. Packaging (D8.91) and envelope (pVSV-G) plasmids were kindly provided by Dr. L. Glushankova (Institute of Cytology of RAS, Russia). The HEK-293T cells were transfected using PEI (polyethylenimine) with a mixture of all three plasmids. The supernatants of the cultures were harvested and concentrated using PEG/NaCl solution [60]. The titers of lentivirus were determined by the formation of colonies of infected cells that were resistant to puromycin. Cells were selected on puromycin (2.0 μg/mL; Sigma, USA) at least 2 weeks prior to the start of experiments.

For the analysis of glioblastoma cell proliferation, a growth curve was plotted for each obtained C6 cell line (C6-wt, C6-shHsp70, C6-shHdj1, C6-shHdj2). The baseline cellular density was 5 × 10^4^ cells/mL. The C6 cell concentration was measured every day with a Neubauer Chamber counting.

### Orthotopic model of rat glioblastoma

Male Wistar rats weighing 250–300 g were purchased from an animal nursery (“Rappolovo” RAMN, St. Petersburg, Russia). Animals were anesthetized before mounting in a stereotactic frame (David Kopf Instruments, Tujunda, CA) with 10 mg “Zoletyl-100” (tiletamine hydrochloride and zolazepam, “Virbac santé Animale”, France) and 0.2 mL, 2% Rometar (xylazinum hydrochloride, “Bioveta”, Czech Republic) intraperitoneally. The C6 glioblastoma cells were inoculated (4 × 106 cells/mL; 15 μL) into the nucl. caudatus dexter. Fifteen animals were assigned to each experimental group (i.e., C6-wt, C6-shHsp70, C6-shHdj1, and C6-shHdj2). The studies were performed in accordance with the ethical standards and according to the Declaration of Helsinki and national and international guidelines and were approved by the institutional ethical committee.

For analysis of the glioblastoma invasion on the twentieth day following cell inoculation, animals were sacrificed and the brains extracted and fixed in 4 % formalin. Brain tumor cryosections were stained with DAPI and rhodamine-phalloidin. Images were obtained using confocal microscopy.

The MRI was acquired using a high-field 11.0 T MR scanner (Bruker, Germany) and a head coil. Anesthesized rats were positioned with the tumor centered on the surface coil. Coronal single-slice spin echo images were selected on orthogonal images through the center of the tumor. Images were obtained using following regimes: TurboRARE-T2 (repetition time [TR]/echo time [TE] 4200/36 ms, flip angle (FA)180°, field of vision (FoV) 2.5 × 2.5 cm, matrix 256 × 256), RARE-T1 (TR/TE 1500/7.5 ms, FA 180°, FoV 2.5 × 2.5 cm, matrix 256 × 256), FLASH (gradient echo) (TR/TE 350/5.4 ms, FA 40°, FoV 2.5 × 2.5 cm, matrix 256 × 256), and a multi-slice multi-echo (MSME) regime. The obtained images were analysed using Analyze software (AnalyzeDirect, Inc., Overland Park, KS).

### Survival analysis

Following injection of the glioblastoma cells for survival analysis, animals were divided into four groups (10 rats each) as follows: (1) C6-wt, (2) C6-shHsp70, (3) C6-shHdj1, and (4) C6-shHdj2. Kaplan-Meier survival curves were plotted for the four experimental groups.

### Western blot

The knock-down of Hsp70, Hdj1 and Hdj2 in C6 cells was verified by Western blot analysis. The C6 cells selected with puromycin (3 × 10^5^) were lyzed on ice in solution containing 20 mM TrisHCl pH 7.5, 20 mM NaCl, 0.01% Triton X-100, 1 mM EDTA, 1 mM PMSF, 10 μg/mL leupeptin, 10 μg/mL pepstatin. Equal amounts of protein (50 μg/lane) were electrophorezed in a 10% SDS polyacrylamide gel. Following transfer to the PVDF membrane non-specific binding was blocked with 5% fat free milk in PBS overnight at +4°C. The membranes were further incubated overnight at 4°C with monoclonal mouse antibodies as follows: J32 antibodies for Hdj1and 3C5 for Hsp70 produced at Institute of Cytology of RAS, KA2A5.6 antibodies for Hdj2 and an anti-N-cadherin antibody (both Abcam, UK).

A loading control was performed via tubulin detection (UBP-bio). Following incubation with primary antibodies, membranes were washed and incubated with horseradish peroxidase-labeled antibodies (Sigma, USA).

### Confocal microscopy

To establish whether the N-cadherin locates of on cell surface C6 cells of all four sub-lines were allowed to settle on cover glass slides and stained with anti Hdj2 antibody (Abcam, UK) on ice, fixed with 4% paraformaldehyde and incubated with secondary anti-rabbit antibody labeled with Alexa488 (Sigma, USA). Nuclei were stained with 4′,6-diamidino-2- phenylindole (DAPI). Fluorescence images were captured with the use of Leica TCS SP2 confocal microscope (Leica, Germany).

### *In vitro* scratch assay

A scratch assay was used to analyze cell motility: 10^5^ C6 cells with down-regulated chaperones were seeded into the wells of a 24-well plate and were cultured overnight. The *in vitro* scratch assay was performed using a sterile 200-μL pipette tip to scratch several straight lines on the cell monolayer. Immediately after scratching, wells of one 24-well plate were washed to remove detached cells with 0.01M phosphate-buffer solution (PBS), and photographed. Cell growth was assessed after 48 h following scratching after the images were captured. The gap distance was measured and the mean was calculated for each cell line.

### The colony-formation test

The colony-formation test was performed in two variants: (1) colony formation by cells from conditioned culture medium containing detached cells that could form colonies on the new substrate. The C6-wt, -shHdj1, -shHdj2 and -shHsp70 cells were seeded into wells of 24-well plates at a concentration of 2 × 10^5^ cells/mL and were allowed to attach to the bottom for 4 h, after which, fresh medium was introduced to remove floating cells and cells were incubated for 18 h. The culture media were collected and transferred to the wells of six-well plates. Twenty four hours later, the colonies formed by the attached cells were fixed with ice-cold methanol, stained with 0.1% crystal violet and forming colonies were calculated using phase-contrast microscopy. (2) Cells of the four C6 sub-lines were seeded in a Boyden camber (8-μm pores) at a concentration of 2 × 10^5^ cells/mL in serum-free medium. The chambers were inserted into wells of a 24-well plate that contained 0.75 mL medium with 10% of FBS for 24 h. Twenty h later, the cells on the outer membrane and the bottom of the plates were fixed and stained as described above and cells and colonies were calculated.

### Spheroid formation and the spheroid invasion test

Ninety-six-well plates were coated with 50 μL 1.5 % agarose and cells were seeded at a concentration of 2 × 10^5^ cells/mL to form spheroids during overnight incubation. To assay the invasion ability, the obtained spheroids were placed on the Matrigel layer in 96-well plates and matrigel was added to the culture medium to form a gel. The spheroids were incubated overnight and were photographed. The size of the spheroids and their invasion edges were determined using the function “Measure” in Image J software.

### Zymography of matrix metalloproteinases

The C6-wt, C6-shHdj1, C6-shHdj2 and C6-shHsp70 cells were seeded into wells of 24-well plates at a concentration of 2 × 10^5^ cells/mL and were cultured in standard conditions overnight. The following day, the wells were washed, the culture medium was replaced with serum-free medium and the cells were cultured for 48 h in standard conditions. The culture media were collected and centrifuged at 300 *g* for 10 min, to remove floating cells and following subsequent centrifugation at 10,000 g for 10 min to remove cellular debris. Then, 50 μL of each medium was mixed with zymography sample buffer (Laemmli buffer without dithiothreitol) and PAGE was performed with gels containing 1 mg/mL gelatin or 0,5 mg/mL casein. The gels were washed twice with 2.5% Triton X-100 for 30 min and were incubated for 12 h in reaction buffer (50 mM Tris-HCl, pH7.6, 0.15 M NaCl, 10 MM CaCl_2_ and 0.05 % Brij 35) and stained with Coomassie blue G-250 to reveal the zones corresponding to MMP-1, MMP-2, MMP-8 and MMP9. The quantitative characteristics of the matrix metalloprotease activity were analyzed using the function “Gels” in Image J software.

### Statistical analysis

The data are generally reported as the mean ± SE. Student's *t*-tests were performed to evaluate differences between the control and treatment groups. Differences were considered to be statistically significant when *p* < 0.05 (*) or *p* < 0.001(**).

## SUPPLEMENTARY FIGURES


