# Supplementary material for: Knock-down of Hdj2/DNAJA1 co-chaperone results in an unexpected burst of tumorigenicity of C6 glioblastoma cells
Source: Oncotarget. 2016 Mar 3;7(16):22050–63. doi: 10.18632/oncotarget.7872 (PMC5008343; doi:10.18632/oncotarget.7872)
Supplement: Supplementary file 1 [file oncotarget-07-22050-s001.pdf]

SUPPLEMENTARY FIGURES

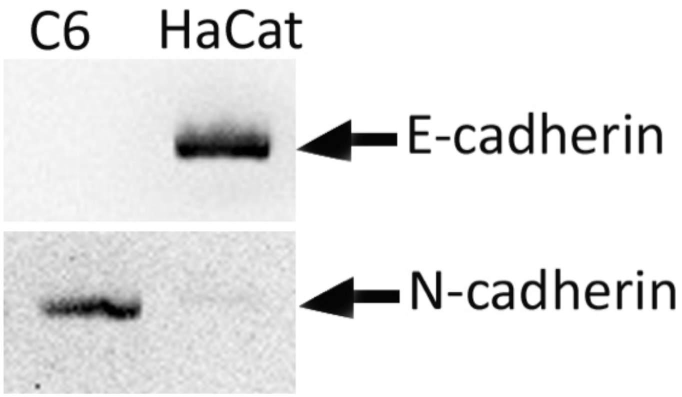

Supplementary Figure S1: C6 glioma cells express N-cadherin but do not express E-cadherin. C6 rat glioma cells and immortolysed human keratinocytes HaCat were subjected to Western blotting analysis and probed with antibody against E- and N-cadherin.

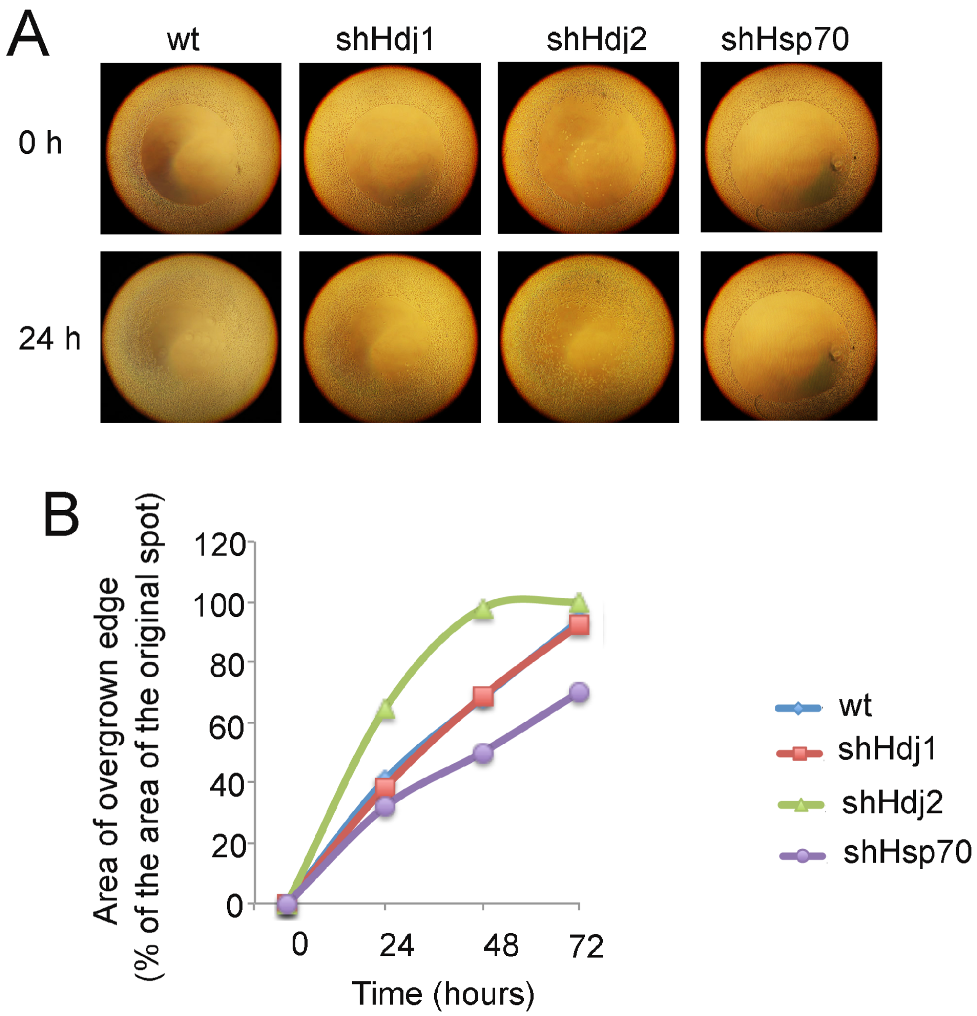

Supplementary Figure S2: Down-regulation of Hdj2 in C6 cells modulates the ability for cell migration as it is shown in wound healing assay. Wound-healing assay was used to analyze cells' motility. 2 µl drops of 1% low melting point agarose solution were placed on the center of the wells of 24-well plates and solidified at +4°C. Cells were seeded in the prepared wells at the concentration 2×10^5 cells/ml and incubated overnight to adhere. The next day culture media was replaced to the fresh one and partly melted agarose drop was removed in the process, so that cell-free spot was formed. Pictures of the spots were taken daily and the size of overgrown area was quantified with ImageJ "Measure" function.

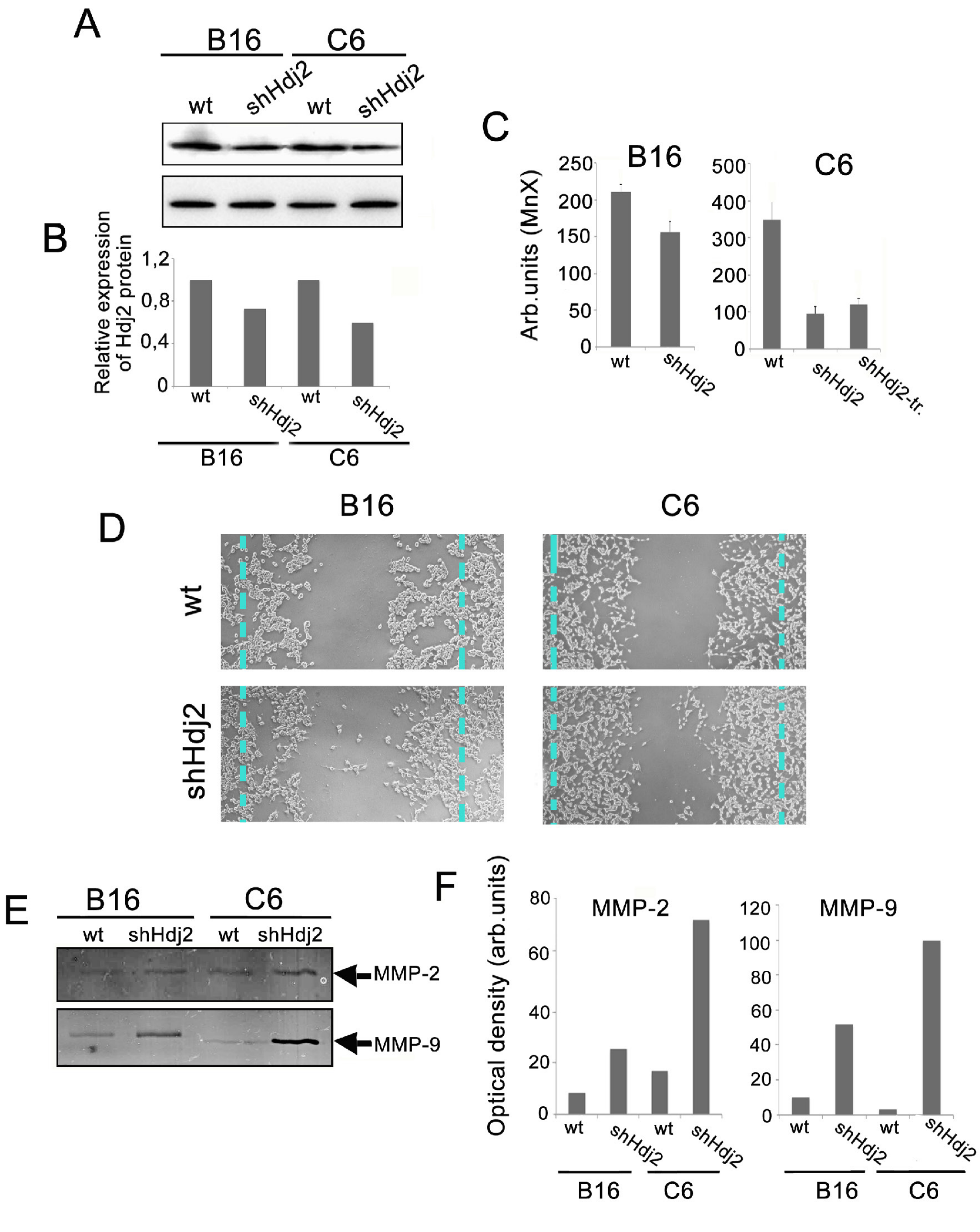

Supplementary Figure S3: Transient infection of rat glioblastoma C6 cells and mouse melanoma cells with lentivirus-encoded shRNA directed against sequences of Hdj2 chaperone leads to increasing of migration rate and metalloproteinases -2 and -9 activity. A. B16 melanoma and C6 glioblastoma cells were infected with lentivirus-encoded shRNA directed against sequences of Hdj2 chaperone (without selection) and supplied for Western blotting with antibody against Hdj2 protein. **B.** The intensity of bands in A was estimated with the use of Image J software. **C.** B16wt and B16-shHdj2 and C6-wt, C6-shHdj2 and C6-shHdj2-tr. transiently infected with shHdj2 were collected, fixed with 4% paraformaldehyde, permeabilized with 0,1% Triton X-100 and stained with anti-Hdj2 antibody. The probes were analyzed with the aid of flow cytometry. **D.** B16 and C6 cells both -wt, and transiently infected with shHdj2 were cultured in six-well plates. A straight scratch was made in individual wells with a 200-µL pipette tip, and the wound was photographed under a microscope after 48 h of incubation (See Materials and Methods for details). **E.** Expression of metalloproteinases MMP-2 and 9 in the culture medium of B16 and C6, -wt, and transiently infected with shHdj2 were defined using zymography (See Materials and Methods for details). **F.** Quantitative analysis of MMP activity was performed using Image J software.
